# Systemic inflammation among adults with diagnosed and undiagnosed cardiometabolic conditions: a potential missed opportunity for cardiovascular disease prevention

**DOI:** 10.3389/fmed.2023.1327205

**Published:** 2024-01-11

**Authors:** Arch G. Mainous, Pooja Sharma, Ara Jo

**Affiliations:** ^1^Department of Health Services Research Management, and Policy, University of Florida, Gainesville, FL, United States; ^2^Department of Community Health and Family Medicine, University of Florida, Gainesville, FL, United States

**Keywords:** National Health and Nutrition Examination Survey (NHANES), cardiovascular disease, inflammation, USA, adults

## Abstract

**Context:**

Systemic inflammation is associated with cardiovascular morbidity and mortality. Since inflammation is not screened in the population, the prevalence, particularly among individuals with undiagnosed cardiometabolic disease, is unclear.

**Objective:**

To assess the prevalence of elevated inflammation using high sensitivity C-reactive protein (hs-CRP) (>0.30 mg/dL) in adults with no cardiometabolic disease, undiagnosed disease and diagnosed disease.

**Methods:**

We conducted a cross-sectional analysis of the 2015–2020 National Health and Nutrition Examination Survey which allows for population estimates of the US population. Adults > = 20 years old were included. HsCRP levels >0.30 mg/dL represented inflammation. Individuals were classified into disease defined as having one or more of the following: diagnosed disease--diabetes, hypertension, hyperlipidemia, or obesity by diagnosis; undiagnosed disease (self-report of no doctor diagnosis but positive biomarker); no disease.

**Results:**

12,946 unweighted individuals representing 315,354,183 adults in the US population were assessed. The proportion of adults with systemic inflammation is 34.63%. The proportion of individuals aged 20 years and older with no disease, undiagnosed disease and diagnosed disease and inflammation was 15.1, 29.1 and 41.8%, respectively. When stratifying by race/ethnicity among individuals with elevated inflammation Non-Hispanic Black people have the highest prevalence (50.35%) in individuals with diagnosed disease followed by Hispanics (46.13%) and Non-Hispanic White people (40.15%) (*p* < 0.01). In logistic regressions adjusted for sociodemographic variables, individuals with undiagnosed cardiometabolic disease have an increased risk of elevated inflammation as measured by CRP (OR 2.38; 95%CI = 1.90–2.99).

**Conclusion:**

In conclusion, a substantial proportion of the adult population, particularly minority and low socioeconomic populations, have elevated inflammation. Systemic inflammation may be a potential focus for disease prevention and disease progression in primary care.

## Introduction

In June 20, 2023, the US Food and Drug Administration approved colchicine as the first anti-inflammatory, atheroprotective cardiovascular treatment (1). The COLCOT trial was a repurposing of colchicine, an anti-inflammatory medication indicated for gout and pericarditis as a secondary prevention for ischemic cardiovascular events among patients who had experienced a myocardial infarction (2). The randomized double-blind placebo controlled trial showed that colchicine led to a significantly lower risk of ischemic cardiovascular events than placebo. In a follow-up randomized double-blind trial among patients with chronic coronary disease, patients on colchicine had significantly lower risk of cardiovascular events than those who received placebo (3). Patients with systemic inflammation, as measured by high sensitivity C-reactive protein (hs-CRP), now have an FDA-approved treatment option demonstrated to reduce the risk of cardiovascular disease by targeting inflammatory pathways.

**Figure 1:**
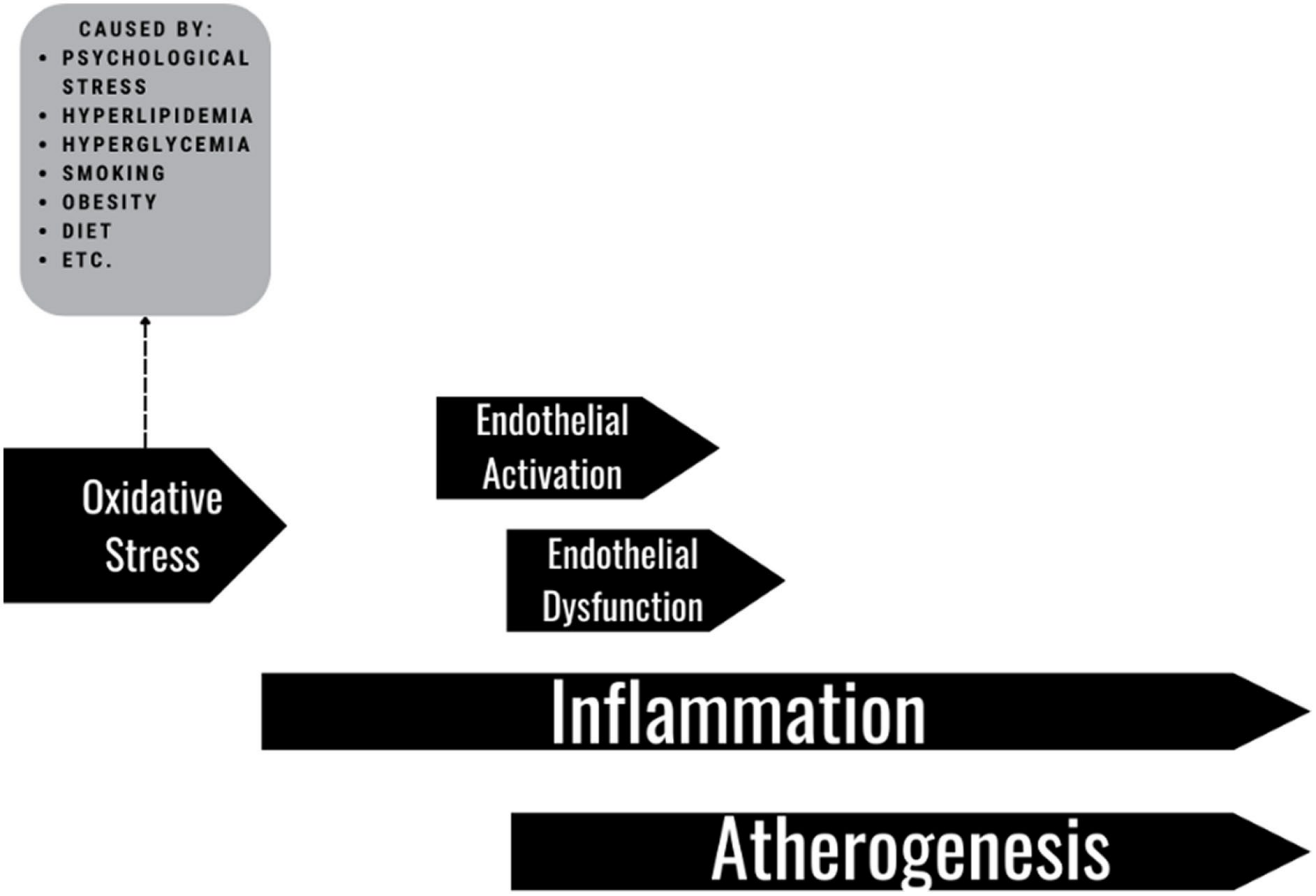
Pathway of pathophysiology of endothelial damage, inflammation and atherogenesis.

Systemic inflammation is associated with the development and progression of many chronic conditions like atherosclerosis, diabetes, hypertension, hyperlipidemia, and obesity, as well as morbidity and mortality (4–6). Figure 1 presents a pathway. Evidence has accumulated indicating the significant relevance of low-grade inflammatory processes to cardiovascular disease, cancer and vascular risk factors (4, 7). Further, hs-CRP is a strong independent predictor of future cardiovascular events (6, 8). Cohort studies have shown that elevated CRP is associated with mortality and cardiovascular disease (CVD) events for patients with various CVD locations like coronary artery disease, cerebrovascular disease, peripheral artery disease, and abdominal aortic aneurysm (9).

Inflammation is a modifiable risk factor. Pro-inflammatory diets are associated with increased CVD, cancer, and mortality risk while anti-inflammatory diets may reduce the risk of CVD outcomes (10, 11). Cigarette smoking is also associated with elevated CRP (12). Several major clinical trials have demonstrated that therapy targeted for lowering inflammation will decrease primary and secondary CVD risk (13–15). A variety of medicines have anti-inflammatory effects (13–15). The recent trial of colchicine as an anti-inflammatory medication that spurred the FDA’s recent ruling on treatment did show a decreased risk for secondary CVD prevention (3).

Although there is considerable evidence of the link between inflammation and cardiometabolic disease development and progression, and inflammation is a modifiable risk factor, inflammation is not typically measured clinically except in certain autoimmune and infectious diseases. It is unclear whether there is a missed opportunity for prevention of systemic inflammation and concomitant CVD in primary care, particularly for patients with undiagnosed cardiometabolic disease. The goal of this study was to examine the prevalence of systemic inflammation in the population and the possibilities of missed opportunities for CVD prevention.

## Methods

In this cross-sectional study, we analyzed the National Health and Nutrition Examination Survey (NHANES) for the years of 2015–2020 prior to Pandemic. The NHANES is a large, nationally representative survey that samples the non-institutionalized population of the United States using a stratified multistage probability sample design. This national, public use, deidentified database is considered “not human subjects” by the Institutional Review Board of the University of Florida.

To account for nationally representative population estimates, the National Center for Health Statistics applies a multilevel weighting system. The survey included a standardized medical examination including blood analysis for examining biomarkers and health-related interviews. The application of weights and variables accounting for the complex survey design allowed us to provide population estimates. Our study focused on adults aged 20 years-old and older.

### Inflammation

HS-CRP was used as an outcome. It was categorized into two levels to indicate inflammation. A threshold of 0.30 mg/dL has demonstrated a significant linkage to the development of cardiometabolic disease. Elevated HS-CRP was defined as >0.3 mg/dL, as recommended by the Centers for Disease Control and Prevention (CDC) and the American Heart Association (AHA) (8).

### Cardiometabolic conditions

Cardiometabolic conditions were identified by self-reported questionnaire. Individuals were classified into three categories: (1) no cardiometabolic condition, (2) one or more diagnosed chronic disease(s) such as diabetes, hypertension, hyperlipidemia, or obesity determined by a doctor, and (3) at least one undiagnosed chronic disease(s) identified by biomarker(s) and a self-reported survey.

### Diabetes

Individuals who had a hemoglobin A1c (HbA1c) level of 6.5% or greater upon examination and who reported never being told by a physician that they had diabetes (excluding gestational) were considered to have undiagnosed diabetes. Individuals who had never been told that they had diabetes and who had an HbA1c level < 6.5% were considered not to have diabetes (16).

### Hypertension

Blood pressure was measured using a sphygmomanometer during a physical examination at a mobile examination center. Three measures of blood pressure were obtained, with a fourth attempt for those who had a previous measurement interrupted or incomplete. The first readings of systolic and diastolic blood pressure were analyzed. Undiagnosed hypertension was defined as having a systolic blood pressure of ≥140 mm Hg or diastolic blood pressure of ≥90 mm Hg for respondents who reported never having been told by a physician that they had high blood pressure or hypertension. This corresponds to the levels recommended by the World Health Organization (17). Individuals who reported never Individuals who reported never being told they had hypertension or high blood pressure who had a systolic blood pressure < 140 mm Hg, and a diastolic blood pressure < 90 mm Hg were considered not to have hypertension.

### Hyperlipidemia

Individuals who had a total cholesterol level of ≥200 mg/dL and who were never told by a physician that they had high cholesterol were considered to have undiagnosed hypercholesterolemia. Individuals who reported never being told they had high cholesterol and who had a total cholesterol level of <200 mg/dL were considered not to have hypercholesterolemia (18).

### Obesity

Individuals who had been told by a physician that they were overweight and had a body mass index (BMI) ≥30 kg/m2 were considered to have been diagnosed with obesity [World Health Organization (19)]. Persons who were never told by a physician that they were overweight and had a BMI consistent with obesity were considered to have undiagnosed obesity. Individuals who were never told by a physician and had BMI <30 were not considered to be obese.

### Covariates

Covariates included demographics and socioeconomic status (SES). Demographics included age, sex and race/ethnicity and SES included education and the poverty-income ratio. The poverty-income ratio was included because of the implications of financial and psychological stress on inflammation. Race/ethnicity was categorized into four groups: (1) Non-Hispanic White, (2) Non-Hispanic Black, (3) Hispanics, and (4) Other.

### Statistical analyses

Descriptive analyses were conducted including ANOVA for mean differences and chi-square tests for categorical variables. Prevalence of elevated hs-CRP was measured, and ANOVA was used to examine significant differences in prevalence across three groups. Unadjusted logistic regression and adjusted regression model controlling age, sex, race/ethnicity, education, and poverty income ratio were used to determine associations between diagnosis status of cardiometabolic disease and elevated hs-CRP. Analyses were conducted using the SAS survey package in version 9.4.

## Results

The total study population was 12,946 representing 315,354,183 individuals. The prevalence of diagnosed cardiometabolic disease(s) and undiagnosed cardiometabolic disease are displayed in Table 1. The prevalence of elevated hs-CRP was highest in diagnosed disease followed by those with undiagnosed diseases and those with no evidence of disease. The proportion of adults with systemic inflammation is 34.63%. The proportion of individuals aged 20 years and older with no disease, undiagnosed disease and diagnosed disease and inflammation was 15.1, 29.1 and 41.8%, respectively. A higher proportion of Non-Hispanic Black people and Hispanics than Non-Hispanic White people exhibited undiagnosed disease.

**Table 1:** Population estimates for demographic characteristics of diagnosed, undiagnosed, and no cardiometabolic disease among adults aged> = 20 years, 2015–2020 (Unweighted *N* = 12,946; Weighted *N* = 315,354,183).

Factors	Diagnosed disease	Undiagnosed disease	No evidence of disease	*p* value
Unweighted sample size	8,640	2086	2,220	–
Weighted population size	203,421,624	52,308,003	59,624,556	–
Weighted prevalence of Elevated hs-CRP (%)	41.77	29.13	15.11	<0.01
Age (years)				<0.01
20–44 years	19.38	47.05	33.57	
45–64 years	73.47	17.05	9.48	
65 and above	86.15	9.87	3.98	
Sex (Male) %	63.28	16.96	19.76	0.17
Race/ethnicity (%)				<0.01
Non-Hispanic White	66.56	15.54	17.90	
Non-Hispanic Black	64.83	15.86	19.31	
Hispanic	58.69	20.51	20.80	
Other	59.99	17.93	22.08	
Education (%)				0.02
High School or Less	64.27	18.27	17.46	
Some College or Graduate	64.62	15.64	19.74	
Poverty Income Ratio (%)				0.01
At/below Poverty Line	59.83	18.76	21.41	
Above Poverty Line	65.79	15.99	18.22	

Among individuals with elevated hs-CRP, Non-Hispanic Black people and Hispanic people had higher proportions than Non-Hispanic White people, particularly among adults with undiagnosed and diagnosed cardiometabolic disease (*p* < 0.01) (Table 2). Education attainment and poverty to income ratio (PIR) showed significant differences in the prevalence of elevated hs-CRP (Table 2, *p* < 0.05). Lower SES populations also showed elevated inflammation. Low education attainment and low PIR accounted for higher prevalence of hs-CRP. Individuals who were at or below the poverty line or those with lower educational attainment showed significantly higher prevalence of elevated hs-CRP, particularly among those with undiagnosed or diagnosed cardiometabolic disease (*p* < 0.05).

**Table 2:** Prevalence of elevated hs-CRP by diagnosed, undiagnosed and no evidence of cardiometabolic disease stratified by sex, race/ethnicity, education and poverty-income ratio.

Factors	Diagnosed disease	Undiagnosed disease	No evidence of disease
Sex (%)			
Male	16.63	12.30	5.91
Female	25.14	16.82	9.20
Race/ethnicity (%)			
Non-Hispanic White	40.15	25.31	14.68
Non-Hispanic Black	50.35	37.82	15.06
Hispanic	46.13	37.61	18.88
Other	36.35	26.62	11.70
Education (%)			
High School or Less	45.59	36.16	17.97
Some College or Graduate	39.63	24.46	13.68
Poverty Income Ratio (%)			
At/below Poverty Line	46.19	38.84	15.84
Above Poverty Line	40.66	25.98	14.87

The percentages in the Table 2 represents the row percentage of proportion of individuals with high hs-CRP across diagnosed, undiagnosed and no evidence of cardiometabolic disease further segmented by sex, race/ethnicity, education, and poverty income ratio.

Table 3 presents the results of the unadjusted and adjusted odds ratio from the logistic regressions. In unadjusted analyses, individuals with undiagnosed cardiometabolic disease were along with those with diagnosed disease to be significantly more likely to show elevated hs-CRP as compared to those with no evidence of disease. In the analyses adjusted for demographic and SES variables, the odds of having increased hs-CRP were increased by 138% for those with undiagnosed disease and were increased by 353% in those with diagnosed disease as compared to normal individuals.

**Table 3:** Unadjusted and adjusted logistic regression model examining the association of elevated hs-CRP and disease diagnosis status.

Disease diagnosis status	Unadjusted Odds Ratio (95% CI)	Adjusted Odds Ratio^**^ (95% CI)
Hs-CRP		
No evidence of disease	1.00	1.00
Undiagnosed	2.31 (1.85–2.88)	2.38 (1.90–2.99)
Diagnosed	4.03 (3.33–4.88)	4.53 (3.65–5.62)

^**^Controlling for Age, Sex, Race/ethnicity, Education and Poverty income ratio.

## Discussion

The study’s findings revealed a significant association between inflammation (hs-CRP) and diagnosed and diagnosed cardiometabolic disease. To our knowledge, this is the first study identifying the significance of elevated hs-CRP among individuals with undiagnosed diseases. These findings emphasize the importance of clinical investigation of hs-CRP in a primary care context for high-risk populations to potentially treat inflammation as a strategy to prevent cardiovascular events and death (20). This evidence further supports the development of patient-centered services in medical practice, there is a need to update guidelines on cardiometabolic diseases with inflammation as a risk factor for better patient outcomes. Non-Hispanic Black people and Hispanic people and individuals with low SES were shown here to have a higher prevalence of inflammation. These individuals are at high risk for cardiovascular morbidity and mortality. Since inflammation is not typically a focus of screening or CVD prevention, it is essential to pay attention to the healthcare needs of these vulnerable people.

Previous studies demonstrated substantial evidence of the link between elevated hs-CRP (as an inflammatory biomarker) and progression of cardiometabolic disease. Several studies have examined that hs-CRP is an established independent risk factor and can predict cardiovascular disease events and its recurrence (8, 9). In accordance with the present results, previous studies have demonstrated that incidence of major cardiovascular events can be reduced by treatment of inflammation (by lowering hs-CRP levels) in undiagnosed individuals (13, 15). However, inflammation is considered a modifiable risk but is not usually measured clinically except for certain autoimmune and infectious diseases. Therefore, screening high-risk populations for elevated hs-CRP and early initiation of anti-inflammatory treatment would contribute to the reduction of the risk of cardiometabolic diseases.

This study has several strengths. The use of the NHANES dataset, which has a complex sampling methodology to provide nationally representative population estimates of the US population, many millions of people, is one of the study’s key strengths. In addition, this study examined the inflammation prevalence among populations with undetected diseases.

There are also several limitations to this study. One of the limitations includes selection of hs-CRP was the primary inflammatory marker we used to identify disease risk. However, although there are other inflammatory markers of chronic diseases, hs-CRP is a standard method to assess the risk of developing cardiometabolic diseases. A second limitation study is that the “Other” racial/ethnic group includes adults self-identified by multiple racial/ethnic designations. Third, the NHANES does not contain information on active infection or malignancies that could significantly raise inflammatory marker levels. Further, there are a variety of variables that we used that are measures of risk factors for cardiovascular disease (e.g., hypertension, hyperlipidemia) but the NHANES does not contain direct measures of clinical cardiovascular disease (e.g., coronary artery calcium, carotid ultrasound). There are many variables that are collected but as a population-based survey the medical history is limited.

To conclude, there exists a missed opportunity for prevention of systemic inflammation particularly for undiagnosed cardiometabolic disease. Systemic inflammation may be useful to assess as a potential focus for disease prevention and disease progression in primary care. Such a focus could have particular benefits among vulnerable populations who are at increased risk for CVD morbidity and mortality.

## Data availability statement

Publicly available datasets were analyzed in this study. This data can be found here: https://wwwn.cdc.gov/nchs/nhanes/search/datapage.aspx?Component=Questionnaire&Cycle=2017-2020.

## Author contributions

AM: Conceptualization, Data curation, Methodology, Writing – original draft, Writing – review & editing. PS: Conceptualization, Formal analysis, Writing – review & editing. AJ: Formal analysis, Writing – review & editing.
